# Learning and Nicotine Interact to Increase CREB Phosphorylation at the *jnk1* Promoter in the Hippocampus

**DOI:** 10.1371/journal.pone.0039939

**Published:** 2012-06-28

**Authors:** Justin W. Kenney, Rachel L. Poole, Michael D. Adoff, Sheree F. Logue, Thomas J. Gould

**Affiliations:** Department of Psychology, Temple University, Philadelphia, Pennsylvania, United States of America; University of California San Diego, United States of America

## Abstract

Nicotine is known to enhance long-term hippocampus dependent learning and memory in both rodents and humans via its activity at nicotinic acetylcholinergic receptors (nAChRs). However, the molecular basis for the nicotinic modulation of learning is incompletely understood. Both the mitogen activated protein kinases (MAPKs) and cAMP response element binding protein (CREB) are known to be integral to the consolidation of long-term memory and the disruption of MAPKs and CREB are known to abrogate some of the cognitive effects of nicotine. In addition, the acquisition of contextual fear conditioning in the presence of nicotine is associated with a β2-subunit containing nAChR-dependent increase in *jnk1* (*mapk8*) transcription in the hippocampus. In the present study, chromatin immunoprecipitation (ChIP) was used to examine whether learning and nicotine interact to alter transcription factor binding or histone acetylation at the *jnk1* promoter region. The acquisition of contextual fear conditioning in the presence of nicotine resulted in an increase in phosphorylated CREB (pCREB) binding to the *jnk1* promoter in the hippocampus in a β2-subunit containing nAChR dependent manner, but had no effect on CREB binding; neither fear conditioning alone nor nicotine administration alone altered transcription factor binding to the *jnk1* promoter. In addition, there were no changes in histone H3 or H4 acetylation at the *jnk1* promoter following fear conditioning in the presence of nicotine. These results suggest that contextual fear learning and nicotine administration act synergistically to produce a unique pattern of protein activation and gene transcription in the hippocampus that is not individually generated by fear conditioning or nicotine administration alone.

## Introduction

Modulation of nicotinic acetylcholine receptors (nAChRs) via acute nicotine administration is known to enhance a number of learning and memory tasks in both rodents and humans with hippocampus dependent tasks being particularly susceptible to modulation [1], [2], [3]. Nicotine administered both systemically and infused directly into the hippocampus results in the enhancement of a variety of hippocampus dependent tasks, such as contextual fear conditioning, trace fear conditioning and spatial object recognition [4], [5], [6], [7] and acute nicotine can enhance synaptic plasticity in the hippocampus [8]. Importantly, infusions of antagonists to high affinity β2-subunit containing nAChRs into the hippocampus prevent the systemic nicotine induced enhancement of contextual and trace fear conditioning [4], [6] suggesting that nicotine acting in the hippocampus is not only sufficient but necessary to enhance learning. Nonetheless, despite these well documented enhancing effects of nicotine on hippocampus-dependent learning and synaptic plasticity, the molecular mechanisms downstream from nAChRs that mediate this enhancement remain largely unexplored.

The mitogen activated protein kinsaes (MAPKs) are known to be important in the regulation of learning, memory and synaptic plasticity [9]. Furthermore, stimulation of nAChRs has been found to regulate various members of the MAPK family. In both the rodent brain and in various *in vitro* neuronal systems, nicotine results in an increase in the phosphorylation of p42/44 MAPK (also known as the extracellular regulated kinase; ERK1/2) [10], [11], [12], [13]. Furthermore, inhibiting MEK (mitogen activated protein kinase kinase), the upstream effector of ERK1/2, prevents the nicotine induced enhancement of contextual fear conditioning [14]. We have also previously found that the c-jun N-terminal kinase 1 (JNK1 also known as MAPK8) gene is upregulated in the hippocampus during the consolidation of a nicotine enhanced contextual fear memory; this effect is mediated through β2-subunit containing nAChRs [15]. Taken together, these data suggest that downstream effectors of ERK may be important for the transcriptional regulation of *jnk1* that is involved in the nicotine-induced enhancement of contextual fear conditioning.

ERK activation can result in an increase in the phosphorylation of cyclic AMP response element binding protein (CREB) in neurons [16]. CREB is a transcription factor integral for the consolidation of long-term contextual fear memories [17], [18], [19], and both fear conditioning and synaptic activity result in an increase in CREB mediated gene transcription [20], [21]. CREB mediated gene transcription is largely thought to be regulated via its phosphorylation at Ser 133 [22], [23] and nicotine has been found to increase CREB phosphorylation both *in vitro* and *in vivo* [13], [24], [25], [26]. Importantly, the nicotine induced increase in CREB phosphorylation has been found to be dependent upon ERK1/2 signaling [13], [24]. However, the transcriptional targets of nicotine induced increases in CREB phosphorylation remain largely unknown. Given the importance of gene transcription in learning and memory in general [27], and that learning and nicotine interact to alter *jnk1* transcription in the hippocampus in particular [15], the present study examined the hypothesis that CREB regulates the transcription of *jnk1* in the hippocampus following the acquisition of contextual fear conditioning in the presence of nicotine.

## Methods

### Subjects and Drugs

Subjects were male C57BL/6J mice 8–12 weeks of age. Mice were group housed (2–4 per cage) with *ad libitum* access to food and water. Mice were maintained on a 12∶12 light:dark cycle (lights on at 07∶00). All procedures were done in accordance with NIH guidelines and were approved by the Temple University Institutional Animal Care and Use Committee.

Nicotine hydrogen tartrate (0.09 mg/kg reported as freebase; Sigma, St. Louis) was dissolved in physiological saline. Either nicotine or saline was administered via intraperitoneal injection five minutes prior to training in contextual fear conditioning; mice that were tested in contextual fear conditioning were also administered nicotine five minutes prior to testing. Dosing based on Gould and Higgins [28].

### Fear Conditioning

Mice were trained in contextual fear conditioning in four identical conditioning chambers (26.5×20.4×20.8 cm) comprised of Plexiglas and housed in sound attenuating boxes (Med Associates, St. Albans, VT). Chamber floors were made of metal rods (0.20 cm diameter) spaced 1.0 cm apart and connected to a shock generator and scrambler (Med Associates, Model ENV-414). Ventilation fans mounted on the side of each box provided background noise and air exchange. Each box was illuminated by a 4 W light bulb from above. Stimuli administration was controlled by a PC running custom programmed LabView software.

For training, mice were placed into the chambers for a total of 5.5 minutes. After an initial 148 second baseline period, mice were administered two 0.57 mA, 2 second footshock unconditioned stimuli (US) separated by a 148 second inter-trial interval. Following the administration of the last footshock, mice remained in the chambers for 30 seconds before being removed. Home-cage control mice were injected with saline or nicotine and handled at the same time points as those mice subjected to fear conditioning but remained in their home-cage (i.e, No FC + Sal or No FC + Nic). This manipulation was chosen as a control given that nicotine is known to enhance the learning of a new context in the absence of US administration [29]. Methods based on Davis et al. [30].

A subset of mice was tested for contextual fear conditioning 24 hours following training. Mice were placed back into the same chambers as during testing and freezing, a species typical fear response, was scored over 5 minutes. Freezing was assessed using a time sampling procedure in which mice were observed for 1 second every 10 seconds and were judged as freezing or active. Data are presented as percent of total 10 second bins in which mice were inactive.

### Chromatin Immunoprecipitation (ChIP)

ChIP was performed as previously described [31] with some modifications. Mice were euthanized via cervical dislocation 30 minutes following training in contextual fear conditioning, or an equivalent amount of time post-injection for home-cage control animals (i.e, No FC + Sal or No FC + Nic). The hippocampus or cerebellum was rapidly dissected, cut into small pieces and cross-linked via exposure to 1% formaldehyde with gentle rocking for 10 minutes at room temperature. Cross-linking was halted via the addition of glycine (200 mM final concentration) for 5 minutes at room temperature. The tissue was then centrifuged at 2.5 kg for 2 minutes at 4C, the supernatant was discarded, and the pellet was washed three times with ice cold phosphate buffered saline containing protease and phosphatase inhibitors (PIs; Pierce Biotechnology, Rockford, IL). Tissue was then homogenized on ice in 1 mL of lysis buffer (10 mM Tris-HCl (pH 8.1), 10 mM NaCl, 1.5 mM MgCl, 0.5% Igepal-CA630) in a glass-glass homogenizer using the tight pestle (Wheaton, Milville, NJ) then centrifuged at 5.5 kg for 5 minutes at 4C and the supernatant was discarded. The nuclear fraction was resuspended via pipetting in 300 uL of nuclear lysis buffer (50 mM Tris-HCl (pH 8.1), 5 mM EDTA, 1% SDS) with PIs and incubated on ice for 10 minutes then stored at –80C until sonication. Chromatin was sheared via sonication using a BioRuptor (Diagnode, Denville, NJ) on high power for 5 minutes (30 seconds on, 30 seconds off) in ice cold water. Following sonication, a small aliquot (5 μL) of sample was removed and analyzed for DNA concentration using a Nanodop (Thermo Scientific, Rockford, IL) and for chromatin fragment size via agarose gel electrophroesis (fragment sizes of approximately 200–800 bps). For each IP 2 μg of DNA was mixed with IP buffer (16.7 mM Tris-HCl (pH 8.1), 1.1% Triton X-100, 0.01% SDS, 167 mM NaCl) with PIs to a 500 μL volume. Prior to the addition of beads and antibody, 5 μL of the IP mix was removed as 1% input. The appropriate amount of antibody or an equivalent amount of rabbit IgG (see *Antibodies* section below) and 20 μL of protein A magnetic beads (Millipore, Billerica, MA) was then added to the IP and incubated overnight with rotation at 4C. Following incubation, the solutions were washed once each with a low salt (20 mM Tris HCl, 150 mM NaCl, 2 mM EDTA, 1% Triton-X, 0.1% SDS), high salt (20 mM Tris HCl, 500 mM NaCl, 2 mM EDTA, 1% Triton-X, 0.1% SDS), LiCl (10 mM Tris HCl, 250 mM LiCl, 1 mM EDTA, 1% deoxycholate, 1% Igepal-CA630) and TE (10 mM Tris HCl, 1 mM EDTA) wash buffers then eluted via incubation at 62C for two hours in elution buffer (0.1 M NaHCO_3_, 1% SDS) and 0.1 µg/µL proteinase K (Invitrogen, Carlsbad, CA). Cross-links were reversed via incubation at 95C for 10 minutes, tubes were then allowed to cool to room temperature and DNA was isolated and purified using QiaQuick spin columns (Qiagen, Valencia, CA) and eluted twice in nuclease free water to a total volume of 200 µL.

### Quantitative Polymerase Chain Reaction (qPCR)

qPCR was performed using 9 µl DNA, 250–500 nM primer solution and 10 µl Fast SYBR Green PCR master mix (Applied Biosystems, Austin, TX). Reactions were carried out in triplicate on the 7500 Fast Real-Time PCR System (Applied Biosystems) using the following conditions: 95C for 20 seconds followed by 40 cycles of 95C for 15 seconds then 60C for 45 seconds. Prior to use in the analysis of DNA obtained from ChIP, primers were checked for efficiency (100±10%) using mouse genomic DNA and specificity using melt curves and size analysis via agarose gel electrophoresis. Melt curves were run following all experiments to ensure primer specificity. Primers used in experiments can be found in table 1.

**Table 1:** Primers for qPCR.

Target	Forward (5′–3′)	Reverse (5′–3′)
jnk1a	CCGCTGTCCCTTTGTCTTG	GCACATCTATTCTGTTCCATACTACC
jnk1b	GGGAGGAGGGGTTAGTGTTT	GAGGCTCGTCAGTTTATCCG
jnk1c	TGTAATGGGAACAGTCTACCTGAA	CTCGCCAACAACGGAGAA
nr4a2	GTGTGAGGACGCAAGGTCTG	CACGACTGGGGCTGATTT
LINE1	AAACGAGGAGTTGGTTCTTTGAG	TTTGTCCCTGTGCCCTTTAGTGA

### SDS-PAGE

The nuclear fraction of protein samples were loaded into 4–20% gradient gels (BioRad, #456–1093) and then transferred to a nitrocellulose membrane (Invitrogen, LC2001). Membranes were washed in Tris-buffered saline (TBS) and blocked in 5% BSA in Tween-20 TBS (0.1%; TBST) for 1 hour at room temperature. Membranes were incubated with anti-pCREB (1∶1000) at 4C with gentle shaking overnight. Membranes were then washed with TBST 3X for 5 minutes at room temperature and incubated with anti-rabbit secondary (1∶2000, Vector, PI-1000) and anti-biotin (1∶2000, Cell Signaling, 7075) for 1 hour. Membranes were washed 3 times for 5 minutes each in TBST and once in TBS, then transferred to a dish containing equal volumes of chemiluminescent substrate (Thermo Scientific, #34080) for 5 minutes with gentle rocking. Membranes were imaged using a Kodak Gel Logic 1500 imaging system and accompanying software.

### Antibodies

Antibodies obtained from Cell Signaling (Danvers, MA) were CREB (9197, 10 μL) and pCREB Ser133 (4276, 10 μL) and the rabbit IgG negative control (2729); antibodies obtained from Millipore were H3-Ac (06–599, 5 μg) and H4-Ac (06–598, 5 μg).

### Data Analyses

Median Ct values from samples run in triplicate were used to analyze the ChIP-qPCR data. ChIP data are presented as either percent input or as a fold change over home-cage control mice (No FC + Sal) as calculated using the ΔΔCt method [31]. Statistical analyses were done using independent samples t-tests or one-way ANOVAs followed by Tukey HSD post-hoc tests or Dunnett's t-tests (with the No FC + Sal as the reference group) where appropriate. Behavioral data was analyzed by independent samples t-test.

## Results

### Analysis of CREB binding to the jnk1 promoter region

**Figure 1:**
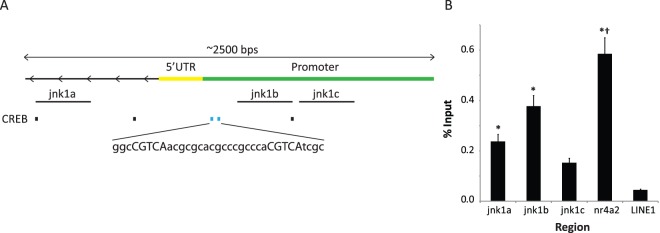
Identification of CREs in the *jnk1* **promoter region.** A) Depiction of the *jnk1* promoter region with an indication of identified CREs. CREs in blue indicate sites conserved between *mus musculus* and *homo sapiens* and capitalized letters indicate half-site CREs. Lines labeled jnk1a, jnk1b and jnk1c indicate genomic regions covered by primers used in qPCR. B) Analysis of CREB binding to the *jnk1* and *nra4a2* promoters and *LINE1* in the hippocampus. There was greater binding of CREB to the *nr4a2* promoter than all other regions examined. The regions covered by the jnk1a and jnk1b primers demonstrate greater CREB binding than *LINE1*. *−p<0.05 compared to *LINE1*; † −p<0.05 compared to jnk1a, jnk1b and jnk1c.

Initial *in silico* analysis of the *jnk1* promoter region was performed using the evolutionary conserved region browser and the TransFac database [32], [33]. This analysis revealed that there are several CREB binding sites in the *jnk1* promoter region, two of which are conserved between *mus musculus* and *homo sapien* (Figure 1A) suggesting that they may be functionally relevant [34]. Additionally, the two identified conserved CREB binding sites are both half-sites (CGTCA) as opposed to the full 8-bp palindromic CRE (TGACGTCA).

CREB binding to the *jnk1* promoter region was analyzed using ChIP (Figure 1B). As a positive control for CREB binding the samples were also analyzed for the presence of a previously characterized palindromic CREB binding site in *nr4a2* [31]. As a negative control, the samples were analyzed for the presence of the long interspersed nuclear element 1 (LINE1) retrotransposon, elements that makes up approximately 20% of the mouse genome [35] but are not typically expressed at high levels due to the accumulation of mutations and repressive epigenetic modifications [36]. qPCR analysis of hippocampi subjected to ChIP indicated that there was a high level of binding of CREB to the CRE site in the *nr4a2* promoter, with somewhat less binding in the *jnk1* promoter region and very little binding to *LINE1* (Figure 1B). A one-way ANOVA revealed a main effect of genetic region (*F*(4,238) = 30.4, p<0.001). Tukey post-hoc tests revealed that binding of CREB to the *nr4a2* promoter was greater than all other regions (p<0.05) and that binding to the genetic regions covered by the primer sets jnk1a and jnk1b were greater than *LINE1* (p′s <0.05). Indeed, this is what we would expect based on the fact that the *jnk1* promoter region contains a weaker half-site CRE whereas the *nr4a2* promoter contains a full palindromic CRE.

### The effects of learning and nicotine on CREB binding to the jnk1 promoter

**Figure 2:**
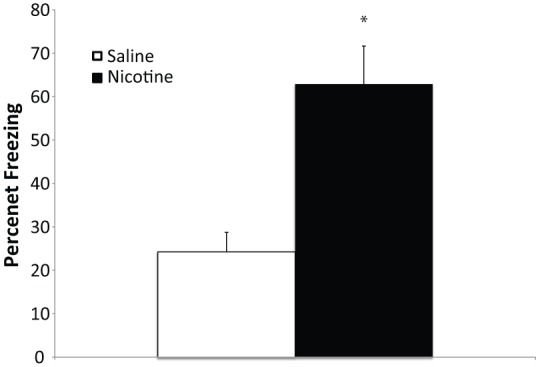
Nicotine enhances contextual fear conditioning. Mice administered 0.09 mg/kg nicotine prior to training and testing demonstrate enhanced contextual fear learning as compared with saline treated animals. * −p<0.05 compared to saline treated mice.

**Figure 3:**
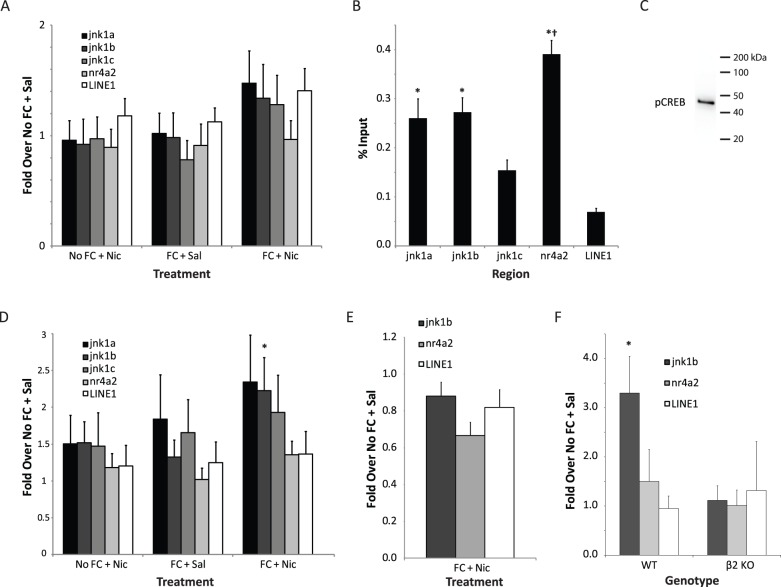
Fear conditioning in the presence of nicotine increases pCREB binding to the *jnk1* promoter in the hippocampus. A) There was no effect of fear conditioning, nicotine administration or fear conditioning in the presence of nicotine on CREB binding to the *jnk1* or *nr4a2* promoter or *LINE1* in the hippocampus. B) Analysis of pCREB binding to the *jnk1* and *nr4a2* promoters and *LINE1* in the hippocampus. There was greater binding of pCREB to the *nr4a2* promoter region than all other regions examined. The regions covered by the jnk1a and jnk1b primers demonstrated greater pCREB binding than *LINE1*.* −p<0.05 compared to *LINE1*; † −p<0.05 compared to jnk1a, jnk1b and jnk1c. C) Immunoblot for pCREB using the same antibody as in the ChIP experiments found that the antibody was specific to pCREB. D) Fear conditioning in the presence of nicotine results in an increase in pCREB binding to the region of the *jnk1* promoter covered by the jnk1b primer set in the hippocampus. * −p<0.05 compared to No FC + Sal group. E) Fear conditioning in the presence of nicotine had no effect on pCREB binding to the *jnk1* promoter in the cerebellum. F) Fear conditioning in the presence of nicotine resulted in an increase in pCREB binding to the *jnk1* promoter in the hippocampus of WT but not β2-subunit nAChR KO mice. * −p<0.05 compared to No FC + Sal group.

The administration of nicotine enhances contextual fear conditioning (Figure 2; *t*(14) = 3.9, p<0.05) and previous work has found that fear conditioning in the presence of nicotine results in an increase of *jnk1* in the hippocampus, but fear conditioning or nicotine administration alone were without effect [15]. Thus, given that CREB binds to the *jnk1* promoter region (Figure 1), we examined whether or not there was any change in CREB binding to the promoter region following contextual fear conditioning in the presence of nicotine. There were no changes in CREB binding to the *jnk1* promoter following fear conditioning, nicotine administration or fear conditioning in the presence of nicotine (Figure 3A). Furthermore, there were no changes in CREB binding to *nr4a2* or *LINE1* following any of the experimental manipulations. CREB is considered to be a constitutively bound transcription factor and its ability to regulate transcription is largely mediated via phosphorylation at Ser133 [23], [37]. Analysis of hippocampi subjected to ChIP using a pCREB antibody found that there were differences in the binding of pCREB to the genetic regions examined (*F*(4,159) = 20.8, p<0.001). Like CREB, pCREB was bound to the CREB binding site in *nr4a2* to a greater extent than all other regions examined, and the regions covered by the jnk1a and jnk1b primer sets demonstrated greater binding than *LINE1* (p′s<0.05) (Figure 3B). In addition, the pCREB antibody was found to be specific to pCREB and not other potentially related proteins as indicated by a single band evident from western blotting (Figure 3C). In the hippocampi of mice trained in the presence of nicotine, there was a main effect of pCREB binding to the jnk1b primer set (F(3, 28) = 2.97, p<0.05) but no effect at any of the other primer sets examined (Figure 3D). Post-hoc Dunnett's t-tests indicated that fear conditioning in the presence of nicotine resulted in an increase in pCREB binding at the site of jnk1b primer set as compared to home-cage control animals (No FC+Sal; p<0.05). There was no change in pCREB binding at the *jnk1* promoter following nicotine alone or fear conditioning alone (p's>0.05). This suggests that CREB phosphorylation may be regulating *jnk1* transcription following learning the presence of nicotine.

To determine if the increase in pCREB binding to the *jnk1* promoter occurred elsewhere in the brain besides the hippocampus, we examined the effect of fear conditioning in the presence of nicotine in the cerebellum, a region that also has high levels of nicotinic expression [38] and in which previous work suggests that learning and nicotine do not interact to alter *jnk1* transcription [15]. We found that there was no change in pCREB binding to the *jnk1* promoter in the cerebellum following learning in the presence of nicotine (p>0.05, Figure 3E).

### Increased binding of pCREB to the jnk1 promoter following learning and nicotine requires β2-subunit containing nAChRs

Both pharmacological and genetic evidence suggests that β2-containing nAChRs are required for the enhancing effect of nicotine on contextual fear conditioning [4], [39], [40]. Furthermore, the increase in *jnk1* mRNA following learning in the presence of nicotine is absent in β2-subunit nAChR KO mice [15]. Thus, we examined the binding of pCREB to the *jnk1* promoter in β2-nAChR KO and WT mice. There was an increase in pCREB binding to the *jnk1* promoter at the region covered by the jnk1b primer set in the hippocampus of WT (*t*(4) = 3.46, p<0.05), but not in β2-nAChR KO mice (p>0.05; figure 3F).

### Learning and/or nicotine do not alter histone acetylation at the jnk1 promoter

**Figure 4:**
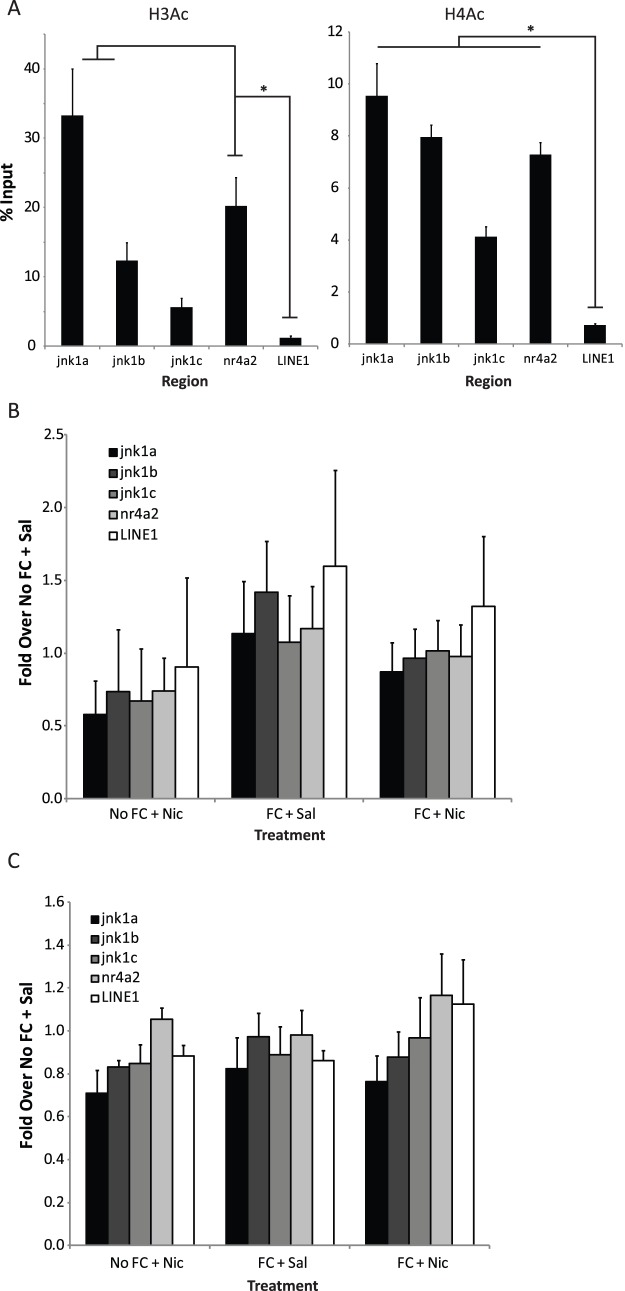
Fear conditioning and/or nicotine administration did not alter histone acetylation at the *jnk1* or *nr4a2* promoter regions. A) Analysis of H3 and H4 acetylation at the *jnk1* and *nr4a2* promoters and *LINE1* in the hippocampus. There were increased levels of both H3 and H4 acetylation at both the *jnk1* and *nr4a2* promoters as compared to *LINE1*. * −p<0.05 as compared to *LINE1*. B) There was no effect of nicotine administration, fear conditioning or fear conditioning in the presence of nicotine on H3 acetylation at the *jnk1* or *nr4a2* promoters or *LINE1* in the hippocampus. C) There was no effect of nicotine administration, fear conditioning or fear conditioning in the presence of nicotine on H4 acetylation at the *jnk1* or *nr4a2* promoters or *LINE1* in the hippocampus.

One result of increased CREB phosphorylation is thought to be recruitment of various transcriptional co-factors, such as CREB binding protein (CBP) and p300 [41], [42], that augment CREB mediated gene transcription and have also been found to be important for learning, memory and synaptic plasticity [43], [44], [45]. These co-factors are thought to enhance transcription, in part, by acting as lysine acetyltransferases [46] at histones H3 and H4, resulting in a more accessible chromatin structure thereby increasing the likelihood of the transcriptional machinery binding to promoter regions [47]. Importantly, changes in histone acetylation have also been implicated in learning, memory and synaptic plasticity [31], [48], [49]. Thus, we examined H3 and H4 acetylation in the hippocampi of mice (Figure 4A). One-way ANOVAs revealed a main effect of genetic region for both H3-Ac (*F*(4,85)  = 22.4, p<0.001) and H4-Ac (*F*(4,85)  = 44.0, p<0.001). Tukey post-hoc tests revealed that H3 acetylation was higher at the region covered by the jnk1a primer set than all other sets examined and that binding at the jnk1b and nr4a2 primer sets were both greater than *LINE1* (p's<0.05). H4 acetylation at the jnk1a set was higher than all other regions examined except jnk1b and the regions covered by the jnk1b, jnk1c and nr4a2 primers were all greater than LINE1 (p's<0.05). However, there was no effect of learning, nicotine or learning in the presence of nicotine on either H3 or H4 acetylation at the *jnk1* or *nr4a2* promoters or *LINE1* in the hippocampus (figure 4B & C). Thus, it is unlikely that alterations in histone acetylation regulate the transcription of *jnk1* following learning in the presence of nicotine.

## Discussion

The findings of the present study indicate that nicotine and learning interact to alter CREB phosphorylation at the *jnk1* promoter region in the hippocampus. The increase in pCREB binding to the *jnk1* promoter is not due to an increase in CREB binding or accompanied by an increase in histone acetylation. Furthermore, as we have demonstrated for the enhancement of learning by nicotine and the nicotine/learning-associated increase in hippocampal *jnk1* transcription [15], [39], [40], the increase in pCREB binding to the *jnk1* promoter following learning in the presence of nicotine requires β2-subunit containing nAChRs. In addition, the increase in pCREB binding to the *jnk1* promoter does not occur throughout the brain, as it is absent in the cerebellum, although this does not rule out the possibility that it is occurring in other brain regions. Taken together, these data suggest that the signaling events that lead to an increase in CREB phosphorylation at the *jnk1* promoter in the hippocampus are important for the mnemonic effects of nicotine on hippocampus dependent learning and memory.

The finding that nicotine may modulate contextual fear learning via CREB phosphorylation is consistent with previous work that has found that CREB is involved in learning, memory and the rewarding properties of nicotine. Genetic disruption of CREB function consistently results in learning and memory deficits in a variety of hippocampus dependent tasks [17], [18], [50], [51]. Furthermore, nicotine administration results in an increase in CREB phosphorylation in the ventral tegmental area (VTA) and nucleus accumbens and the rewarding effects of the drug have been found to require CREB activation in the nucleus accumbens (Walters et al., 2005; Brunzell et al., 2009). *In vitro*, nicotine has been found to result in an increase in pCREB via ERK signaling in both dissociated hippocampal neurons and PC12 cells (Nakayama et al., 2001; Hu et al., 2002). In ciliary ganglion neurons, nicotine administration results in an increase in pCREB via both ERK and calmodulin kinase II/IV (CaMK) signaling pathways (Chang and Berg, 2001). Thus, the findings from the present study are in strong agreement with the previous literature and significantly extend it by being the first study to find a specific gene that may be regulated by nicotine through a change in CREB phosphorylation.

Implicating CREB phosphorylation in the effects of nicotine on learning and memory suggests the involvement of various signaling molecules given that CREB is known to be phosphorylated via the ERK, PKA, CaMKIV and p38 MAPK pathways [52]. Thus, there are two possibilities for how learning and nicotine may interact to increase CREB phosphorylation at the *jnk1* promoter: 1) nicotine and fear conditioning are acting on the same pathway or 2) nicotine and fear conditioning are acting on two separate pathways that converge on CREB and both must be activated for the memory enhancing effects of nicotine. In support of the first interpretation, the enhancement of contextual fear conditioning by nicotine has been found to be dependent upon ERK [14] and the action of nicotine at high affinity nAChRs in the hippocampus is able to reverse learning deficits induced by NMDA glutamate receptor antagonists [53]. The action of glutamate at NMDARs in the hippocampus and ERK activation are both known to be integral to contextual fear learning [19], [54], [55] and blocking NMDARs also prevents ERK activation in the hippocampus during learning, in hippocampal slices during LTP stimulation, and in primary cell culture in response to glutamate [56], [57], [58], [59]. ERK can phosphorylate CREB via the activation of p90 ribosomal S6 kinase (RSK) and mitogen and stress activated protein kinase (MSK) [60], [61]. It may be the case that learning and nicotine both independently activate the ERK pathway and are additive in their eventual effect on CREB phosphorylation at the *jnk1* promoter. Alternatively, in support of the second interpretation that nicotine and fear conditioning may be acting via parallel pathways, the fact that neither nicotine alone nor fear conditioning alone are sufficient to alter either *jnk1* transcription [15] or CREB phosphorylation at the *jnk1* promoter, suggests that nicotine may be acting on a hitherto yet unidentified pathway or one that is not typically recruited during fear conditioning. It may be the case that nicotine acts in a permissive fashion that allows learning to engage additional mechanisms resulting in a greater overall response. Delineating between these two interpretations of how nicotine modulates learning is an important goal of future research.

Changes in gene transcription due to CREB activity are largely thought to be regulated via phosphorylation at Ser133 as CREB is considered to be constitutively bound to CRE sites throughout the genome, although there is some conflicting evidence. Binding studies performed in PC12 cells suggest that increasing CREB phosphorylation at Ser133 does not alter its affinity for DNA [22], however, data from hepatoma cells using DNA footprinting and bandshift assays suggest that protein kinase A (PKA) activation of CREB alters binding at various half and palindromic CRE sites [62]. In the present study, there was an increase in pCREB, but not total CREB, binding to the *jnk1* promoter in response to learning and nicotine administration, suggesting that CREB phosphorylation is not regulating the binding of CREB to the *jnk1* promoter in this context. In contrast, Walters and colleagues [25] found that a single dose of nicotine by itself results in an increase in CREB binding to the μ -opiod receptor gene promoter in the ventral tegmental area. While it is not altogether clear what specifically may be responsible for whether or not CREB is constitutively bound at any particular promoter region, differences in magnesium ion concentration [63], basal levels of PKA [62] or DNA methylation [64], [65] may play a role. Thus, there appears to be considerable diversity in the regulation of CREB binding to various promoter regions, perhaps reflecting the particular composition of the cellular milieu at any given time.

One important implication of the present work is that the identification of CREB binding sites in the *jnk1* promoter may provide candidate regions for identifying polymorphisms that contribute to the cholinergic contribution of the pathology of various cognitive disorders that involve nAChR function such as Alzheimer's disease, schizophrenia and addiction [66], [67]. The information obtained in the present study using ChIP allows for the approximate determination of the likely CREB binding site in the *jnk1* promoter. Using evolutionary conservation analysis (Ovcharenko et al., 2004), two highly conserved half-CRE sites immediately upstream from the 5′UTR in the *jnk1* promoter were identified. CREB binding was greatest, and the increase in pCREB binding due to learning and nicotine was significant, at the region covered by the jnk1b primer set. The jnk1b primer set was the closest of the sets used in the present study to the conserved CRE sites (see Figure 1), which strongly suggests that these conserved binding sites are the most likely candidates for the potential regulation of *jnk1* transcription via increases in CREB phosphorylation. Thus, it may be the case that polymorphisms at these conserved CREB binding sites may play a role in mediating the cognitive effects of nicotine given that genetic variability is known to modulate the effects of nicotine in both mice and humans [68], [69].

Taken together, the findings from the present study implicate CREB phosphorylation in the regulation of *jnk1* transcription in the hippocampus following learning in the presence of nicotine. The increase in CREB phosphorylation is not accompanied by an increase in CREB binding to the *jnk1* promoter or an increase in histone acetylation in the promoter region, suggesting that the chromatin at the *jnk1* promoter is likely in an open state and poised for initiating increased transcription. Further work is required to determine how nicotine may be modulating upstream signaling cascades to interact with those stimulated by contextual fear conditioning. The CREB regulated *jnk1* transcription in response to learning and nicotine may be important for the effects of nicotine on cognition and the modulation of cholinergic deficits observed in various disease states.
